# Interleukin‐18, IL‐18 binding protein and IL‐18 receptor expression in asthma: a hypothesis showing IL‐18 promotes epithelial cell differentiation

**DOI:** 10.1002/cti2.1301

**Published:** 2021-06-26

**Authors:** Davinder Kaur, Latifa Chachi, Edith Gomez, Nicolas Sylvius, Christopher E Brightling

**Affiliations:** ^1^ Department of Respiratory Sciences Institute for Lung Health NIHR Biomedical Research Centre University of Leicester Leicester LE1 7RH UK; ^2^ Genomic Core Facility Department of Genetics University of Leicester Adrian Building, University Road, G23 Leicester LE1 7RH UK

**Keywords:** asthma, epithelium, IL‐18, IL‐18BP, IL‐18Rα, IL‐18Rβ

## Abstract

**Objective:**

In asthma, genome‐wide association studies have shown that interleukin‐18 (IL‐18) receptor 1 gene (*IL‐18R1*) and sputum IL‐18 are increased during exacerbations. However, the role of the IL‐18 axis in bronchial epithelial function is unclear. To investigate IL‐18, IL‐18 binding protein (BP) and IL‐18R expression in bronchial biopsies and sputum samples from patients with asthma, and to determine its functional role using *in vitro* bronchial epithelial cells.

**Methods:**

The expression of IL‐18, IL‐18BP and IL‐18Rα was examined in subjects with asthma and healthy controls in bronchial biopsies by immunohistochemistry and IL‐18 and IL‐18BP release in sputum. In epithelial cells, the mRNA and protein expression of IL‐18, IL‐18BP, IL‐18Rα and IL‐18Rβ was assessed by qPCR, flow cytometry, Western blotting and immunofluorescence respectively. IL‐18 function in epithelial cells was examined by intracellular calcium, wound repair, synthetic activation and epithelial differentiation changes.

**Results:**

In biopsies from subjects with asthma, the IL‐18 expression was not different in the lamina propria compared with controls but was decreased in the epithelium. In contrast, the IL‐18BP was decreased in the lamina propria in asthma and was absent in the bronchial epithelium. IL‐18 was released in sputum with IL‐18BP elevated in patients with asthma. The IL‐18Rα expression was not different between health and disease. *In vitro,* IL‐18‐stimulated bronchial epithelial cells increased intracellular calcium, wound repair, metabolic activity, morphological changes and epithelial cellular differentiation.

**Conclusion:**

In asthma, the dynamic interaction between IL‐18, its cognate receptor and natural inhibitor is complex, with differences between airway compartments. Upregulation of IL‐18 can promote epithelial activation and cellular differentiation.

## Graphical abstract

In this study, we demonstrated that the expression of IL‐18 was significantly lower in bronchial epithelium in subjects with asthma than in healthy controls. IL‐18 treatment of human epithelial cells induced metabolic activity, wound repair and epithelial cell differentiation, which could potentially contribute to airway remodelling.

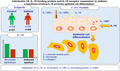

## Introduction

Asthma is a chronic respiratory disorder of the airways characterised by inflammation, variable airflow obstruction and airway wall remodelling.^1^, ^2^ In addition, the number of mast cells localised in the airway smooth muscle (ASM) bundle is increased in asthma and is related to the degree of airway hyper‐responsiveness.^3^ Airway remodelling involves a number of structural changes, including airway wall thickness, volume, subepithelial fibrosis, increased ASM mass, goblet/mucus hyperplasia and altered deposition/composition of extracellular matrix.^4^ As the first line of defence, the pulmonary epithelium forms a barrier between the immune system and the external environment. Evidence suggests that the epithelium plays a critical role in promoting mucosal inflammatory response^5^ and is important in epithelial–mesenchymal transition (EMT).^6^, ^7^

Interleukin‐18 (IL‐18) cytokine, known as the interferon‐γ‐inducing factor, is a product of inflammasome (as is IL‐1β), which is activated by pathogens, environmental factors or host‐derived danger signals.^6^ There is increasing interest in the inflammasome and targeting inflammasome in airway diseases and chronic inflammatory conditions^8^, with IL‐18 playing a role in downstream activation of the inflammasome.

IL‐18 binds to the IL‐18 receptor (IL‐18R). IL‐18R is encoded by two accessory protein genes *IL‐18R1* and *IL‐18R*, which encode an extracellular signalling subunit (IL‐18Rα) and the accessory protein signal‐transducing subunit (IL‐18Rβ) respectively.^9^ Both subunits are required for functional IL‐18 signalling.^9^ Large genome‐wide association studies have consistently identified *IL‐18R1* as one of the loci most strongly associated with asthma.^10^, ^11^, ^12^ IL‐18 is increased in sputum and blood samples during asthma exacerbations.^13^ In cases of fatal asthma, post‐mortem biopsies have shown increased levels of IL‐18 in the epithelium and ASM, compared with control samples.^14^ IL‐18 contributes to airway inflammation with promotion of T helper type 2 responses^15^, ^16^; induction of IgE,^17^, ^18^ IL‐4 and IL‐13, and histamine release in basophils.^18^ IL‐18 binding protein (IL‐18BP) is an endogenous antagonist with neutralising affinity to IL‐18 preventing its interaction with its cell surface receptor inhibiting the IL‐18 biological activity.^19^ In asthma, the interplay between IL‐18, IL‐18BP and IL‐18R^20^, ^21^ is poorly understood and whether the IL‐18 axis impacts epithelial repair or EMT‐associated cell protein or gene expression is unknown.

We hypothesised that bronchial epithelial cells undergo cellular differentiation and EMT cell protein/gene expression changes following IL‐18 stimulation. To test our hypothesis, we investigated IL‐18, IL‐18BP and IL‐18Rα expression in bronchial biopsies and quantified sputum cytokine release in asthma and healthy controls. To further define the role of IL‐18, we determined IL‐18 effects on cell calcium release, wound repair, metabolic activity, proliferation, and morphological and EMT‐associated cell protein expression changes *in vitro* using human primary bronchial epithelial cells and a human virus‐transformed normal bronchial epithelial (BEAS‐2B) cell line.^22^ In addition, we have studied the expression of IL‐18, IL‐18BP, IL‐18Rα, IL‐18Rβ and functionality in several *in vitro* model cells providing a further insight into the role of IL‐18. The expression was explored in the human lung epithelial NC1‐H292 (H292) cell line, in HMC‐1 immature mast cell line, in primary isolated human lung mast cells (HLMCs) and in primary ASM cells.

## Results

### IL‐18, IL‐18BP and IL‐18Rα expression in asthma

**Figure 1:**
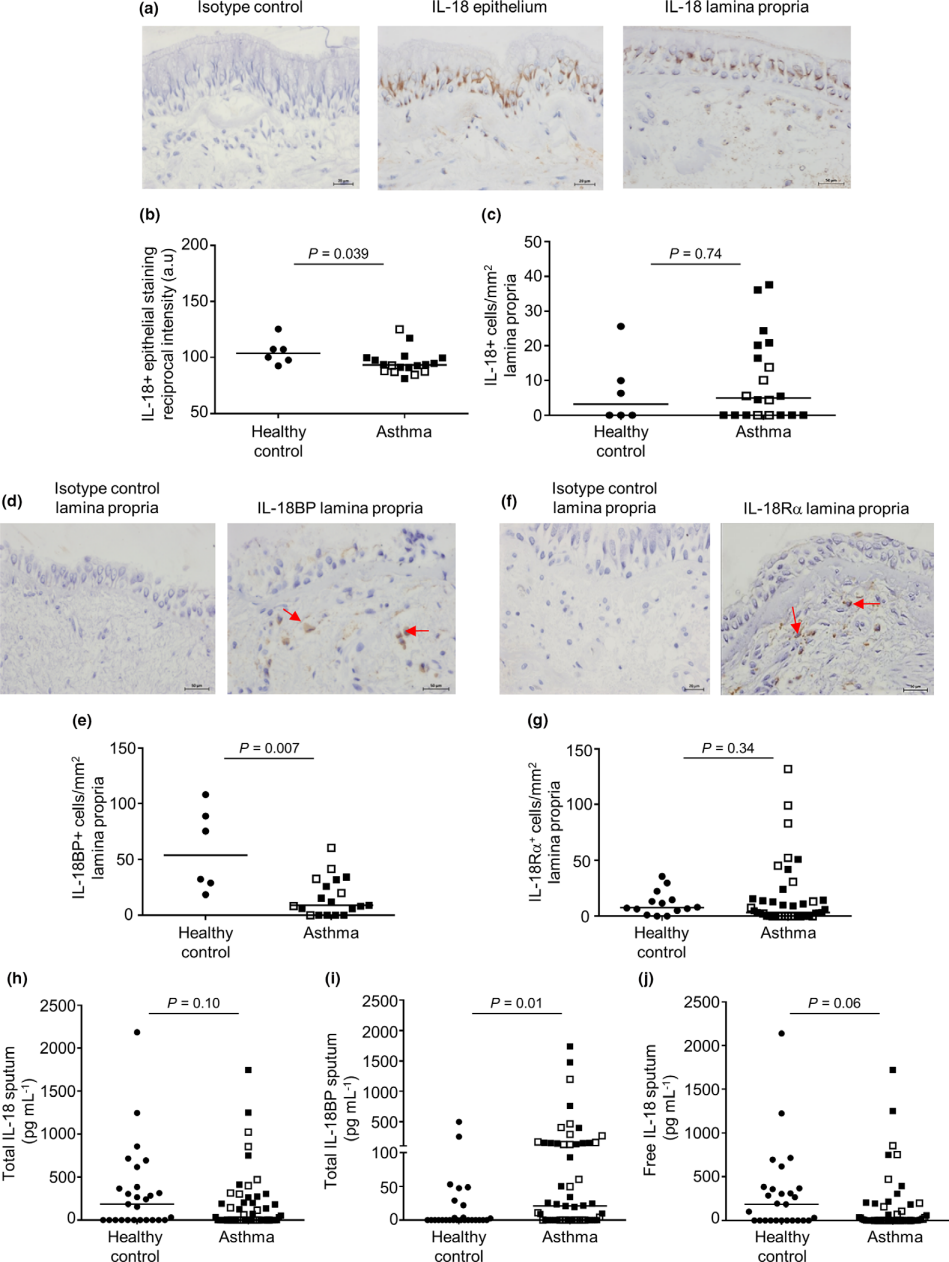
Bronchial biopsy IL‐18, IL‐18BP and IL‐18Rα expression and release in human sputum. **(a)** Representative photomicrograph of normal biopsy specimen illustrating isotype control (magnification ×400), IL‐18 staining within the epithelium (magnification ×400) and lamina propria (magnification ×400). **(b)** IL‐18 epithelial reciprocal intensity staining area under curve (a.u) (*n* = 6 control versus *n* = 18 asthmatic patients) and **(c)** IL‐18‐positive cells mm^−2^ within the lamina propria (*n* = 6 control versus *n* = 20 asthmatic patients). **(d)** Representative photomicrograph of normal subject illustrating an isotype control, IL‐18BP staining within the lamina propria (×400 magnification). **(e)** Cell counts mm^−2^ for IL‐18BP within the lamina propria‐positive cells (*n* = 6 control versus *n* = 19 asthmatic patients). **(f)** Representative photomicrograph of normal biopsy illustrating isotype control and IL‐18Rα‐stained cells within the lamina propria (magnification ×400). **(g)** IL‐18Rα staining cells mm^−2^ within the lamina propria (*n* = 14 control versus *n* = 42 asthmatic patients). **(h)** Total IL‐18, **(i)** IL‐18BP and **(j)** free IL‐18 in healthy controls (*n* = 27) and asthmatic patients (*n* = 58). The concentration of total IL‐18 and IL‐18BP was measured in sputum: the amount of biologically active, free IL‐18 in sputum was calculated as described previously.^33^ ● _=_ healthy control; □ _=_ mild/moderate asthma; and ■ _=_ severe asthma. Horizontal bars represent median, *P* < 0.05, unpaired non‐parametric *t*‐test.

The clinical characteristics of subjects who provided bronchial biopsies are shown in Table 1. Representative photomicrographs of the IL‐18 expression within the epithelium and lamina propria of a bronchial biopsy are displayed in Figure 1a. The expression of IL‐18 was not detected within the ASM bundle. The IL‐18 epithelial reciprocal intensity staining was decreased in subjects with asthma compared with healthy controls (*P* = 0.039, Figure 1b). There was no difference in the IL‐18‐positive cells/mm^2^ within the lamina propria between asthma and health (*P* = 0.74, Figure 1c).

**Table 1:** Clinical characteristics of asthmatic patients and healthy controls

	Bronchial biopsies			Sputum samples		
	Healthy control (*n* = 14)	Mild–moderate asthma (*n* = 18)	Severe asthma (*n* = 24)	Healthy control (*n* = 27)	Mild–moderate asthma (*n* = 24)	Severe asthma (*n* = 34)
Age, years^a^	51 (36–60)	43.5 (32.3–56.8)	51 (47.2–60)	56 (49.5–66.5)	58 (46.7–68)	56.5 (46.2–61.7)
Gender: female/male, *n*	4/10	9/9	17/7	11/16	9/15	23/11
ICS dose	0	Low or medium (GINA 1–3)	High (GINA 4–5)	0	Low or medium (GINA 1–3)	High (GINA 4–5)
Pre‐BD FEV_1_% predicted^b^	97.9 ± 13.8	77.1 ± 28.9	77.25 ± 24.6	101.3 ± 14.3	85.9 ± 17	84.8 ± 21.9
Pre‐BD FEV_1_/FVC^b^	82.5 ± 7.3	70.8 ± 10.1	66.6 ± 13.69	79.1 ± 19.7	76 ± 15.5	72 ± 16.7
% sputum neutrophils^a^	59 (25–6 3)	32.8 (14.6–71.5)	48 (40–67.7)	50 (40.2–65.1)	65 (49.1–86.7)	76 (41.2–89.4)
% sputum eosinophils^a^	0.42 (0.06–0.9)	3.1 (0.3–7.5)	5.25 (0.43–25.38)	1 (0.2–0.9)	1 (0.3–5.4)	2 (0.3–9.1)

ICS, inhaled corticosteroids, FEV_1_, forced expiratory volume in 1 second; FVC, forced vital capacity; pre‐BD, prebronchodilator.

Clinical characteristics of subjects from which tissues for immunohistochemistry were derived and sputum samples for ELISA (IL‐18 and IL‐18BP) analysis.

^a^ Median (interquartile range).

^b^ Mean ± SD.

A representative photomicrograph of the IL‐18BP expression in cells embedded within the lamina propria bronchial biopsy is shown in Figure 1d and in ASM cells in Supplementary figure 1a. The IL‐18BP lamina propria was decreased in subjects with asthma compared with healthy controls (*P* = 0.007, Figure 1e) and between mild moderate and severe asthma groups (*P* = 0.047) but not in ASM cells (Supplementary figure 1b). The IL‐18BP expression was not present within the bronchial epithelium.

A representative photomicrograph of the IL‐18Rα expression within the bronchial biopsy lamina propria cells is shown in Figure 1f. There was no difference observed in IL‐18Rα staining‐positive cells mm^−2^ within the epithelium (*P* = 0.71, data not shown) and lamina propria (*P* = 0.34; Figure 1g) between asthma and health. The IL‐18Rα expression was not identified within the ASM bundle.

The clinical characteristics of the subjects who provided induced sputum are shown in Table 1. Induced total sputum IL‐18 (Figure 1h), IL‐18BP (Figure 1i) and calculated free IL‐18 (Figure 1j) levels were detected both in patients with asthma and in healthy controls. There was no difference between total and free IL‐18 release in the induced total sputum samples from patients with asthma compared with healthy controls. Nevertheless, high levels of total IL‐18BP (*P* = 0.01; Figure 1i) were identified within the asthmatic subjects.

### IL‐18, IL‐18BP and IL‐18Rα expression in epithelial cells

No difference was identified in the level of IL‐18, IL‐18BP and IL‐18R expression in human primary bronchial epithelial cells between asthmatic and healthy controls in all studies; therefore, data were combined throughout.

**Figure 2:**
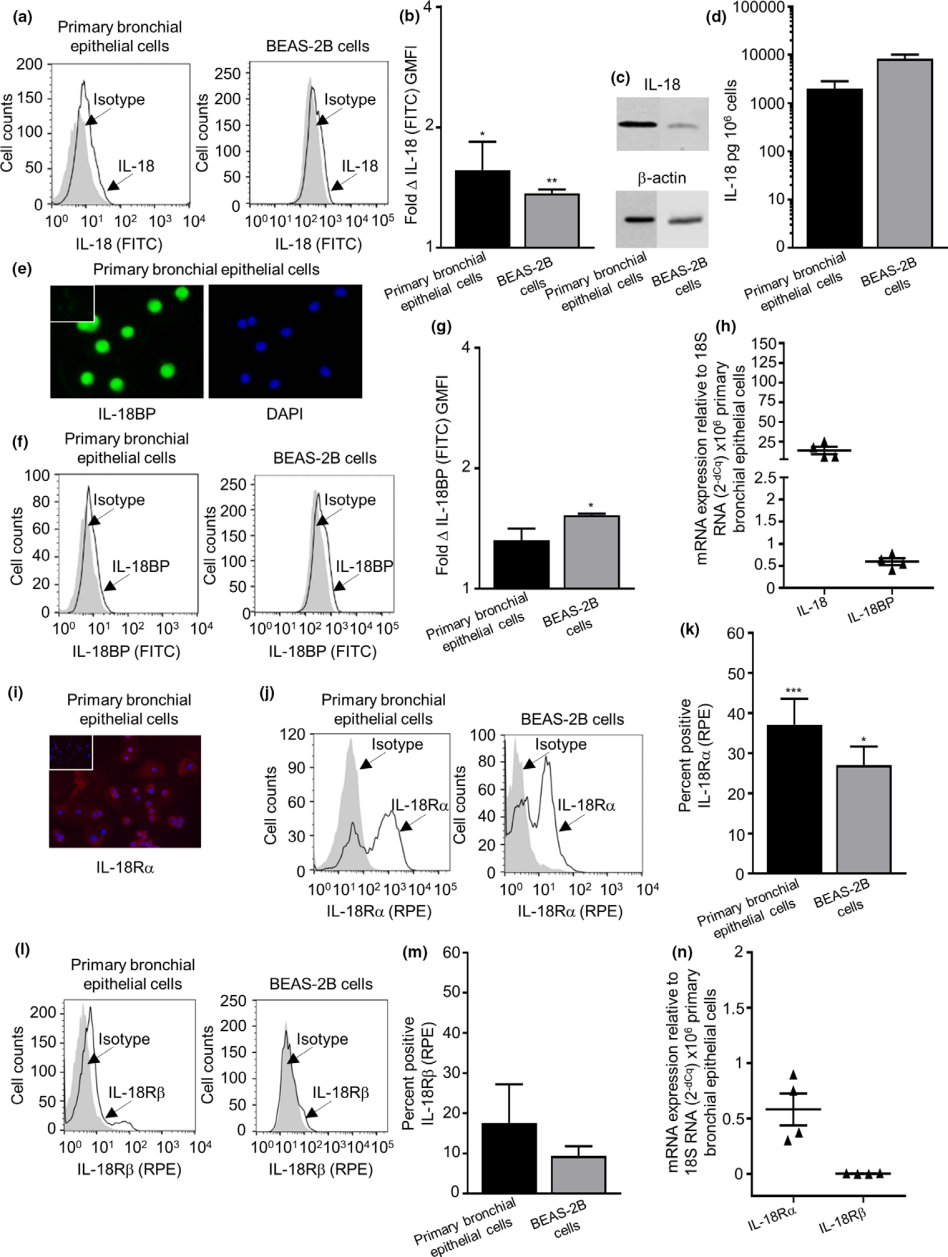
IL‐18, IL‐18BP and IL‐18 receptor expressed by human epithelial cell. **(a)** Representative fluorescent histograms of IL‐18 and **(b)** quantification of total IL‐18 in primary bronchial epithelial cells (*n* = 9) and BEAS‐2B cells (*n* = 5). **(c)** Western blot analysis of IL‐18 in primary bronchial epithelial cells (*n* = 3) and BEAS‐2B cells (*n* = 4, upper gel), and β‐actin was used as a loading control (lower gel). **(d)** IL‐18 spontaneous release after 24 h in epithelial cell supernatants (*n* = 4 or 5). **(e)** IL‐18BP expression in primary bronchial epithelial cells was confirmed by immunofluorescence (nuclei stained blue), IL‐18BP (stained green) and isotype control shown as insert (magnification ×400 *n* = 3). **(f)** Representative fluorescent histograms of IL‐18BP and **(g)** quantification of total IL‐18BP in primary bronchial epithelial cells (*n* = 13) and BEAS‐2B cells (*n* = 5). **(h)** IL‐18 and IL‐18BP mRNA expression analysed by qPCR in primary bronchial epithelial cells. **(i)** IL‐18Rα expression in primary bronchial epithelial cells was confirmed by immunofluorescence (nuclei stained blue), IL‐18Rα (stained red) and isotype control shown as insert (magnification ×200 *n* = 3). **(j)** Representative fluorescent histograms of IL‐18Rα and **(k)** quantification of surface IL‐18Rα in primary bronchial epithelial cells (*n* = 8) and BEAS‐2B cells (*n* = 3). **(l)** Representative fluorescent histograms of IL‐18Rβ and **(m)** quantification of surface IL‐18Rβ in primary bronchial epithelial cells (*n* = 3) and BEAS‐2B cells (*n* = 3). **(n)** IL‐18Rα and IL‐18Rβ mRNA expression was analysed by qPCR in primary bronchial epithelial cells. Data are presented as mean ± SEM. Statistical differences were assessed using the paired or unpaired *t*‐test, *P < 0.05, **P < 0.01 and ***P < 0.001 (versus isotype control).

The IL‐18 expression was identified in primary bronchial epithelial cells, BEAS‐2B cell line, a lung epithelial H292 cell line, HLMC isolated from lung, a human immature mast cell line HMC‐1 cell and primary ASM cells by flow cytometry (Figure 2a and b, Supplementary figure 2a and b) and Western blotting [Figure 2c (original Western blot for IL‐18 is shown in Supplementary figures 3 and 2c)] for all cell types except for ASM cultures. IL‐18 was spontaneously released from primary bronchial epithelial cells, BEAS‐2B cells (Figure 2d) and H292 cells (data not shown).

The IL‐18BP expression was detected in primary bronchial epithelial cells, ASM cells, HLMCs and HMC‐1 by immunofluorescence (Figure 2e and Supplementary figure 2d). The total IL‐18BP expression was detected by flow cytometry in all cells except for HLMCs (Figure 2f, g and Supplementary figure 2e and f). There was no spontaneous release of IL‐18BP in primary bronchial epithelial cells and BEAS‐2B cells. Notably, IL‐18BP was spontaneously released from ASM and H292 epithelial cells (Supplementary figure 2g).

The *IL‐18* gene mRNA and *IL‐18BP* gene mRNA were found to be expressed in 6 out of 6 primary bronchial epithelial cell donors. To further support the presence of IL‐18 and IL‐18BP, mRNA analysis was performed as shown in Figure 2h (and in Supplementary figure 2h and i for all other cell types).

IL‐18Rα expression was identified in primary bronchial epithelial cells (Figure 2i), ASM cells, HLMCs and HMC‐1 cells by immunofluorescence (Supplementary figure 6a). IL‐18Rα subunit expression was identified in primary bronchial epithelial cells, BEAS‐2B cells (Figure 2j and k), ASM, H292 cells, HLMCs and HMC‐1 cells (Supplementary figure 6b and c), and the IL‐18Rβ subunit was evident in all cell types (Figure 2l and m, Supplementary figure 6d and e) by flow cytometry. The *IL‐18R1* gene was found to be present in 6 out of 6 primary bronchial epithelial cell donors, but the *IL‐18R* accessory protein gene was found absent in all donors. To further support the presence of *IL‐18R1* gene, mRNA analysis was conducted as shown in Figure 2n (Supplementary figure 3f and g for all other cells).

To understand the broader effect of IL‐18, we investigated the transcriptional response of primary bronchial epithelial cells (*n* = 6) and ASM cells (*n* = 5) in the presence and absence of IL‐18 by using microarray. No genes were identified in both the primary bronchial epithelial cells and ASM cells post‐IL‐18 stimulation (data not shown).

### Functional responses of epithelial cells to IL‐18

**Figure 3:**
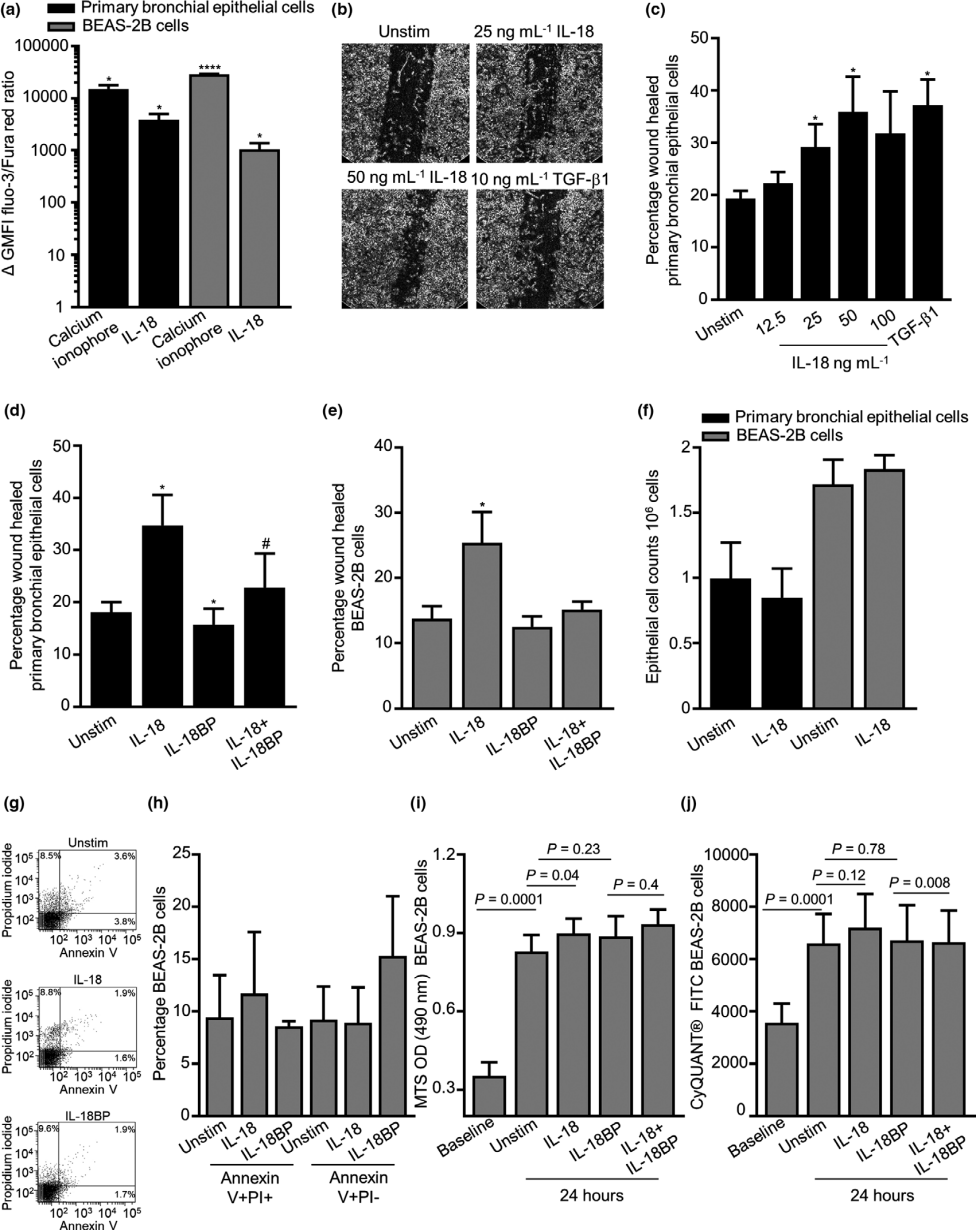
Functional responses by IL‐18 in epithelial cells. **(a)** Calcium flux in primary bronchial epithelial cells and BEAS‐2B cells in the presence of calcium ionophore or IL‐18 (100 ng mL^−1^, *P*‐value compared with baseline Δ GMFI, *n* = 4–6). **(b)** Representative wound repair pictures of primary bronchial epithelial cells after 24 h ± IL‐18 and positive control TGF‐β1 (×100 magnification). **(c)** Concentration‐dependent migration of primary bronchial epithelial cells towards IL‐18 or TGF‐β1 (10 ng mL^−1^, *n* = 6 or 7). **(d)** Percentage wound repair by primary bronchial epithelial (*n* = 4–7) and **(e)** BEAS‐2B cells (n = 6) ± IL‐18 (50 ng mL^−1^) or ± IL‐18BP (150 ng mL^−1^) for 24 h. **(f)** Epithelial cell counts ± IL‐18 over 24 h (*n* = 4‐5). **(g)** Representative dot plots of Annexin V^+^/PI^+^ dot plots, illustrating differential effects of IL‐18 and IL‐18BP in BEAS‐2B cells after 24‐h stimulation. **(h)** Percentage of Annexin V^+^/PI^+^ and Annexin V^+^/PI^−^ cells ± IL‐18 or IL‐18BP for 24 h (*n* = 4). **(i)** Metabolic activity following 24‐h exposure ± IL‐18 or IL‐18BP in BEAS‐2B cells using the MTS assay (*n* = 16), and **(j)** cell proliferation was determined using the CyQUANT® assay (*n* = 16). Data are presented as mean ± SEM. Statistical differences were assessed using the paired or unpaired *t*‐test, *P < 0.05, ***P < 0.001, # compared with IL‐18BP, *P* < 0.01.

IL‐18 triggered Ca^2+^ flux with increased intracellular calcium in primary bronchial epithelial cells and BEAS‐2B cells, as indicated by an increase in Fluo‐3/Fura Red ratio with a maximum response at 100 ng mL^–1^ (Figure 3a). Epithelial wound repair was promoted by exogenous IL‐18 in a dose‐dependent manner in primary bronchial epithelial cells with maximum effect at 50 ng mL^–1^ (Figure 3b and c). The wound healing response of primary bronchial epithelial cells was dramatically reduced by the addition of IL‐18BP alone and in the presence of exogenous IL‐18 over 24 h (Figure 3d). Interestingly, IL‐18 induced wound repair in the BEAS‐2B cell line (*P* = 0.02, Figure 3e) but had no effect on the epithelial H292 cells (Supplementary figure 6a and b).

IL‐18 had no effect on primary bronchial epithelial and BEAS‐2B cell counts (Figure 3f) nor on percentage viability (data not shown). IL‐18 had no effect on epithelial BEAS‐2B cell death and apoptosis as demonstrated by annexin/PI staining (Figure 3g and h). BEAS‐2B cells assessed by the MTS assay demonstrated an increase in cell metabolic activity after 24 h in the presence of exogenous IL‐18 (Figure 3i), and in contrast, this had no effect on cell proliferation as measured by the CyQUANT^®^ assay (Figure 3j). Neutralising IL‐18 in the presence of IL‐18BP had no effect on cell metabolic and proliferative activity over 24 h (Figure 3i and j).

IL‐18 had no effect on ASM cell metabolic activity, wound repair, contraction (Supplementary figure 6a–d) and histamine release in HLMC (Supplementary figure 6e). ASM cells (*n* = 8), HLMCs (*n* = 4) and HMC‐1 cells (*n* = 3) released no cytokine or chemokine in the mesoscale analysis platform following IL‐18 or IL‐18BP incubation (10–100 ng mL^–1^ 24 h, data not shown) compared with untreated cells (data not shown).

### Cell differentiation changes in epithelial cells to IL‐18

EMT is defined to be changes in cell protein expression with markers such as E‐cadherin, α‐smooth muscle actin (SMA, myofibroblast/ASM marker), fibronectin and collagen I (extracellular matrix proteins). To gain further insight into the effects of IL‐18 on cellular differentiation and EMT epithelial cell phenotypic changes, we investigated cell expression levels of E‐cadherin, α‐SMA, fibronectin and collagen I.

**Figure 4:**
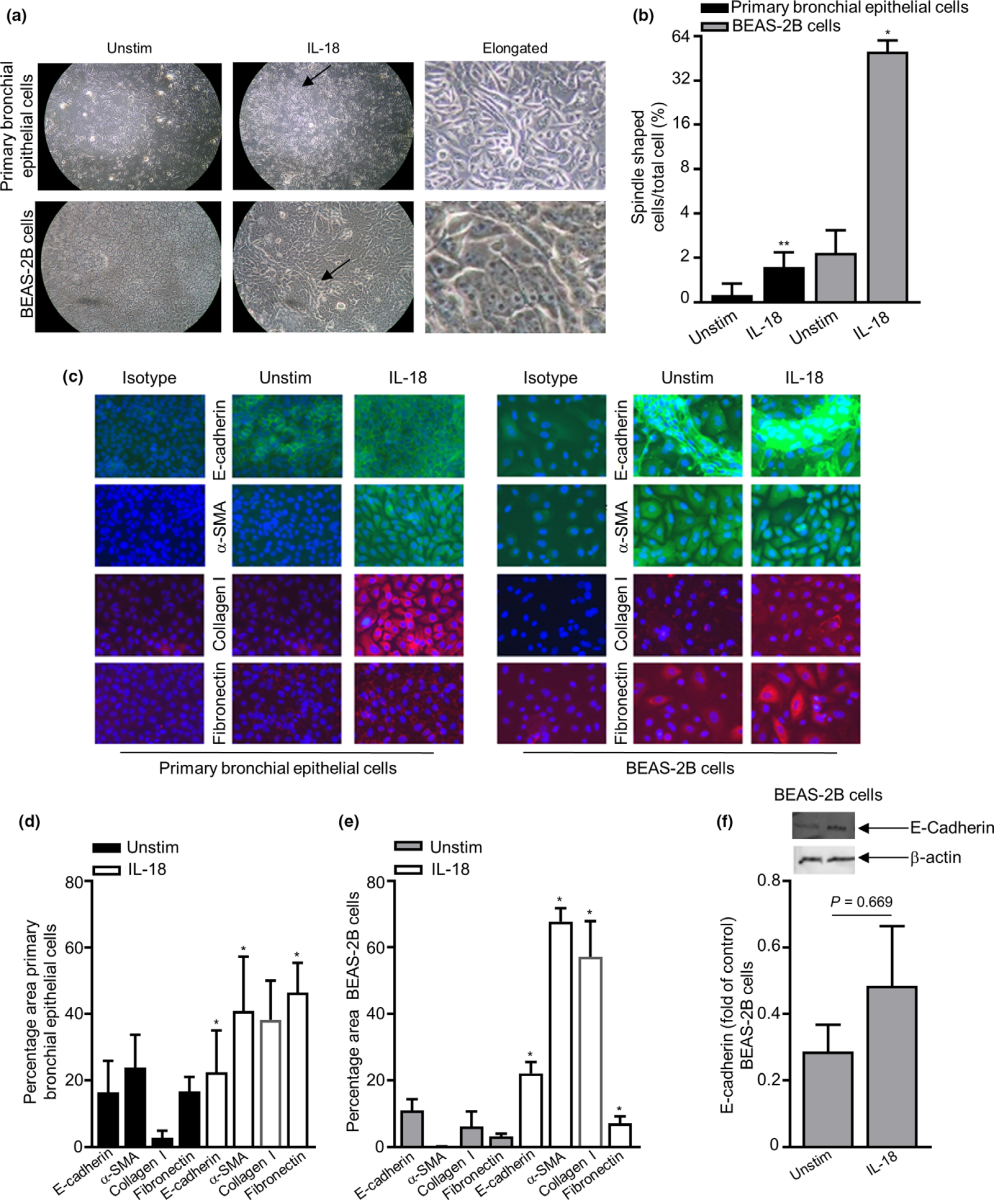
Induction of IL‐18 protein changes in epithelial cells. **(a)** Representative images of primary bronchial epithelial cells and BEAS‐2B cells cultured ± IL‐18 (50 ng mL^−1^) over 24 h (×100 magnification). The black arrow indicates the area of detailed morphology of elongated epithelial cells. **(b)** Percentage quantification of spindle‐shaped cells ± IL‐18 over 24 h was calculated as described in the Methods (*n* = 4). **(c)** Representative immunofluorescence staining of primary bronchial epithelial cells and BEAS‐2B cells (×400 magnification) showing negative isotype control, ± IL‐18 over 24 h for E‐cadherin (stained green), α‐SMA (stained green), collagen I (stained red) and fibronectin (stained red, nuclei stained blue). Quantification of E‐cadherin, α‐SMA, collagen I and fibronectin for **(d)** primary bronchial epithelial (*n* = 3–5) **(e)** and BEAS‐2B cells (*n* = 3–7) calculated as described in the Methods. **(f)** Expression levels of E‐cadherin in BEAS‐2B cells ± IL‐18 over 24 h. A representative blot is shown ± IL‐18 (upper gel) for E‐cadherin, and β‐actin was used as a loading control (lower gel, *n* = 6). The bar chart shows quantification of protein levels compared with β‐actin (*n* = 6). Data are presented as mean ± SEM. Statistical differences were assessed using the paired or unpaired *t*‐test, *P < 0.05 and **P < 0.01 compared with unstimulated (unstim) cells.

**Figure 5:**
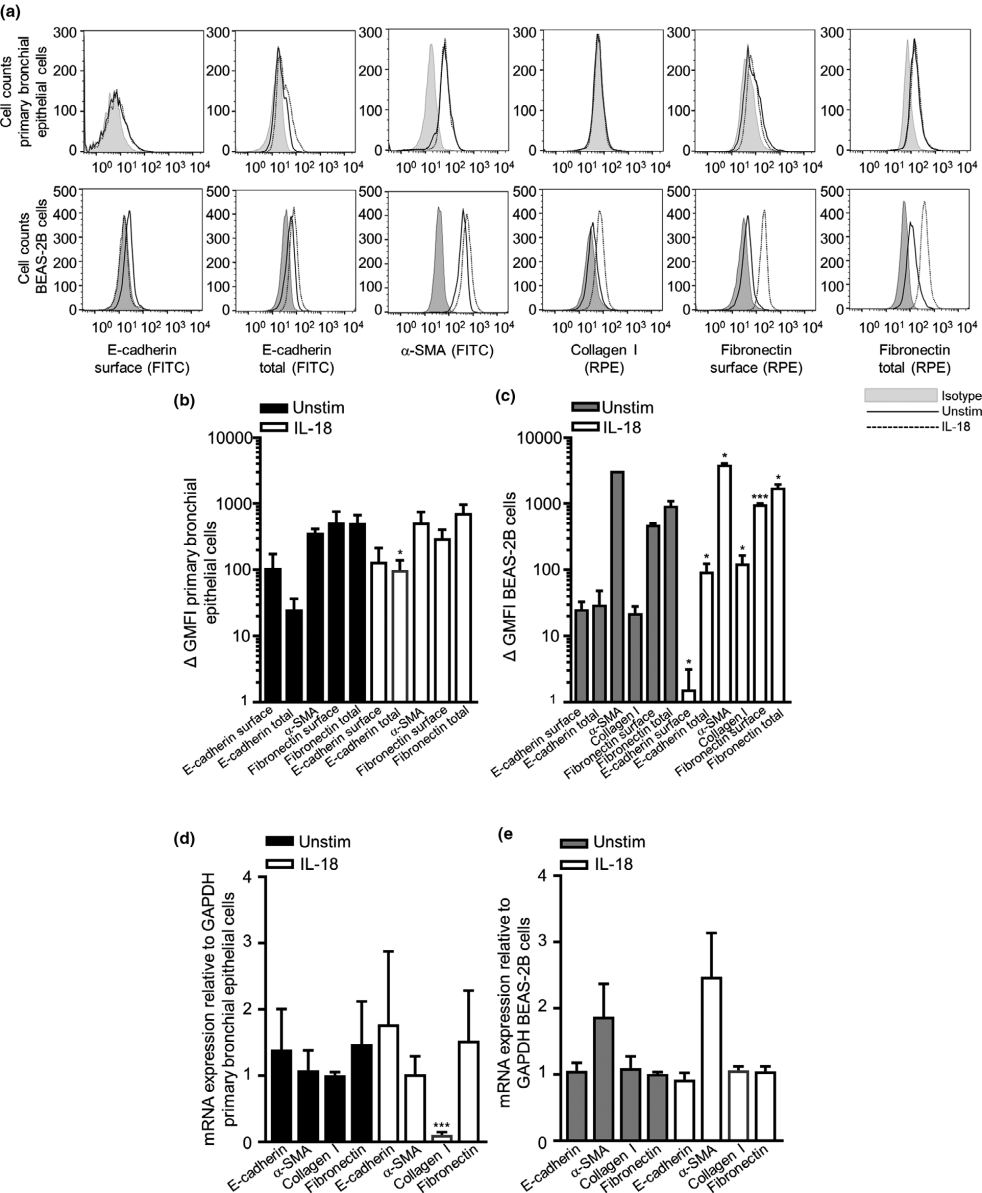
Expression changes in epithelial cells upon IL‐18 treatment. **(a)** Representative fluorescent histogram of primary bronchial epithelial, BEAS‐2B cell surface, total E‐cadherin, α‐SMA, collagen I and surface and total fibronectin ± IL‐18 over 24 h. **(b)** Quantification of E‐cadherin, α‐SMA, collagen I and fibronectin for primary bronchial epithelial (*n* = 3–6) and **(c)** BEAS‐2B cells (*n* = 4–7). **(d)** Relative mRNA expression of E‐cadherin, α‐SMA, collagen I and fibronectin in primary bronchial epithelial (*n* = 3) and **(e)** BEAS‐2B cells (*n* = 9) ± IL‐18 over 24 h by qPCR. Data are presented as mean ± SEM. Statistical differences were assessed using the paired or unpaired *t*‐test, *P < 0.05 and ***P < 0.001 compared with unstim cells.

Semiquantitative assessment of bronchial epithelial cell and BEAS‐2B cell morphological changes showed that cells in the unstimulated control group displayed a cobblestone‐like appearance, the typical feature of epithelial cells. After IL‐18 challenge, the cells underwent morphological changes as observed by EMT cells, where the cells became more elongated, spindle‐like cells (Figure 4a and b). Immunofluorescence staining and quantitative analyses showed that the total expression of E‐cadherin, α‐SMA, collagen I and fibronectin significantly increased in both the primary bronchial epithelial and BEAS‐2B cells (Figure 4c–e). Western blot protein analysis suggested a slight increase in total E‐cadherin in BEAS‐2B cells (Figure 4f, original western blot analysis for E‐cadherin is shown in Supplementary figure 7). Interestingly, only total E‐cadherin expression was substantially increased at the protein level in primary bronchial epithelial cells by flow cytometry, and as for the BEAS‐2B epithelial cell line, surface E‐cadherin was downregulated post‐IL‐18 stimulation and total E‐cadherin, α‐SMA, collagen I, surface fibronectin and total fibronectin were notably upregulated (Figure 5a–c). E‐cadherin, α‐SMA, collagen I and fibronectin were all detected at a gene level in both primary bronchial epithelial cells and BEAS‐2B cells (Figure 5d and e). However, only collagen I was downregulated at a gene level post‐IL‐18 stimulation in primary bronchial epithelial cells (Figure 5d).

## DISCUSSION

We demonstrated IL‐18, IL‐18BP and IL‐18R expression in the bronchial submucosa and *in vitro* in primary bronchial epithelial cells and BEAS‐2B cells. IL‐18 stimulation promoted epithelial cell calcium activation and wound repair, and increased cell metabolic activity, cellular differentiation, and morphological and EMT‐associated protein expression changes. This supports our hypothesis that IL‐18 mediates the early changes in airway remodelling observed in the asthmatic lung via epithelial cell wound repair and EMT‐associated cell protein expression changes by causing epithelial cell differentiation into fibroblast/myofibroblast/ASM‐like cells.

In asthma, the dynamic interaction between IL‐18, its cognate receptor and natural inhibitor is complex with differences between airway compartments. Consistent with previous reports, we found that IL‐18 expression was high in the bronchial epithelium.^23^ In subjects with stable asthma, the expression of IL‐18 was decreased compared with healthy controls. We found that the IL‐18 antagonist IL‐18BP, which neutralises IL‐18 by preventing its interaction with the cell surface receptor,^19^ was present in ASM and mast cells but absent in bronchial epithelium. In sputum, IL‐18BP was increased in stable asthma with a non‐significant decrease in free IL‐18 in sputum in asthma compared with healthy controls. Importantly, in earlier reports IL‐18 in blood and sputum was found to be increased markedly during asthma exacerbations.^13^ This perhaps suggests that IL‐18 might play a key role following airway infection whilst at lower levels during stable asthma.

*In vitro*, we found that IL‐18 stimulation promoted epithelial cell calcium activation and wound repair and increased cell metabolic activity, and morphological and EMT‐associated cell protein expression changes. IL‐18BP inhibited IL‐18‐mediated epithelial wound repair consistent with effects of IL‐18BP inhibiting EMT changes in the murine lung fibrosis model following bleomycin‐induced injury.^24^ In contrast to its impact on epithelial function, we were unable to demonstrate ASM wound repair, proliferation and contraction following IL‐18 stimulation.^25^ Taken together, these findings support our hypothesis that IL‐18 might promote airway remodelling in asthma via effects on epithelial repair, induction of cellular differentiation and EMT protein expression changes.

One possible criticism of this study is that it is likely that the expression of IL‐18 and IL‐18BP in the airway is dynamic, which cannot be captured in a cross‐sectional study. Indeed, the expression was heterogeneous across different airway compartments and likely to change over time and in response to exacerbations. Thus, further studies to determine repeatability in stable disease and response to natural insults and interventions are required.

Several limitations of this study should be discussed. Firstly, the use of mono‐cell epithelial cultures of human primary epithelial cells, immortalised BEAS‐2B epithelial cell line and the H292 epithelial cell line is the limitation as the cells being in a single monolayer of epithelium may not represent a fully differentiated state of the epithelium lacking the airway multilayer barrier and the important air surface liquid (ALI) secreted by human lung epithelial cells. The ALI *in vitro* cell model systems represents a fully differentiated epithelium with more than 1 cell type (ciliated cells, goblet cells and basal cells). We also observed that the BEAS‐2B immortalised epithelial cells seemed to differentiate more in response to IL‐18 than primary bronchial epithelial cells did. This could be because of the primary epithelial cells being in a more basal epithelial phenotype and donor variability. Some of the epithelial cell culture donors used in this study were revived from liquid nitrogen and then cultured, which could have caused the cells to lose their original phenotype and therefore not respond like asthmatic cells would do. If we were to use fresh bronchial brushes for all the studies and allowed the epithelial cells to fully differentiate to ALI cultures, this may have been a robust way of investigating IL‐18 in relation to asthma. Secondly, the ASM donors were a combination of bronchial biopsies, ASM bundles from lung resection material and cells revived from liquid nitrogen cultures. All these factors may have contributed to donor variability in relation to asthma and responsiveness to IL‐18. Thirdly, the use of mast cells in this study, the HMC‐1 cell line that is widely used to exhibit key characteristics of human mast cells such as the expression of histamine, tryptase and the CD117 receptor on the cell surface, has limitations as it lacks the FcεRI receptor making them an immature mast cell type to study in relation to asthma. The HLMCs that were studied were isolated from tissue obtained from patients undergoing lung resection for lung cancer and were not directly from asthmatic patients. Fourthly, one of the main fundamental hallmarks of EMT is loss of cell‐to‐cell adhesion leading to the downregulation of E‐cadherin. We showed that there was no difference in E‐cadherin surface expression by flow cytometry in primary bronchial epithelial cells post‐IL‐18 treatment. On the contrary, we continuously showed increased expression of E‐cadherin in primary bronchial epithelial cells and in the BEAS‐2B cells. The reason for this could be that 24 h was not enough time for IL‐18 to fully exert its EMT changes, and therefore, studies for 48–72 h need to be warranted for future work. Another reason for the internalisation of E‐cadherin could be that IL‐18 may activate trafficking of E‐cadherin by endocytosis recycling E‐cadherin in and out of cells causing loss of cell–cell contact.^26^ This could again be down to the 24‐h stimulation studies, and if we were to increase the IL‐18 activation time, we would potentially demonstrate downregulation of E‐cadherin.

IL‐18 is highly expressed by the airway epithelium and in previous reports was increased in airway samples during an exacerbation. There is continuous interest in inflammasome targeting in airway diseases – what we do not understand at present is the specific roles of IL‐18. Would targeting IL‐18 alone be the approach or would it be more beneficial to target other inflammatory cytokines such as the rest of the IL‐1 family? IL‐1β has been reported to be elevated in a subgroup of asthmatic patients with neutrophilic inflammation.^27^ This may suggest that IL‐18 on its own may cause some morphological changes within the bronchial epithelial cells but may work more efficiently in combination with IL‐1β. Alternatively, IL‐18 may be beneficial in targeting a subgroup of asthmatic patients with specific clinical characteristics.

In conclusion, in asthma the interactions between IL‐18, its receptor and its natural inhibitor IL‐18BP are complex with differences between airway compartments. Upregulation of IL‐18 can promote epithelial activation, differentiation, repair and EMT‐associated cell protein expression. The complex balance between the elements of the IL‐18 axis suggests that although IL‐18 might play a role in airway remodelling in asthma, this mechanism is likely to be quiescent in stable disease, whereas its role following an exacerbation might be more important. This possible dynamic role for IL‐18 in the pathogenesis of asthma warrants further investigation.

## METHODS

### Subjects

Subjects who underwent endobronchial biopsies were recruited from the Glenfield Hospital, Leicester, UK. Asthma was diagnosed based on the presence of typical symptoms and evidence of variable airflow obstruction or airway hyper‐responsiveness and severity according to the Global Initiative for asthma treatment steps (mild–moderate asthma GINA 1‐3, severe asthma GINA 4‐5).^1^ Healthy controls had no respiratory symptoms and presented normal lung function. All current smokers were excluded, and subjects had < 10 pack‐year smoking history. Subjects underwent extensive clinical characterisation including sputum induction^28^ and fibre‐optic bronchoscopy as described previously.^3^ The study was approved by the Leicestershire Ethics Committee, and all patients gave their written informed consent (08/H0406/189).

### Cell culture

Human primary bronchial epithelial cells were obtained from both nasal and bronchial brushings from bronchi and grown onto collagen (Advanced BioMatrix, Carlsbad, CA)‐coated 12‐well plates in bronchial epithelial growth medium (Lonza Pharma & Biotech, Basel) including cell basal growth supplement SingleQuot BulletKit (Lonza), 0.3% fungizone antimycotic (Thermo Fisher Scientific, Leicester) and 1% antibiotic–antimycotic solution (Thermo Fisher Scientific) for 2–7 days. The primary bronchial epithelial cells were then expanded onto collagen‐coated 75‐cm^2^ flasks and replenished with fresh medium three times a week.

Pure ASM bundles were isolated from human bronchial biopsies and lung resection material. Primary ASM cells were maintained in Dulbecco’s modified Eagle’s medium (DMEM) with GlutaMAX‐1 (Thermo Fisher Scientific) supplemented with 10% foetal bovine serum (FBS, Thermo Fisher Scientific), 1% antibiotic–antimycotic solution, 1% nonessential amino acids (Thermo Fisher Scientific) and 1% sodium pyruvate (Sigma‐Aldrich, Gillingham, Dorset). ASM cells were characterised by flow cytometry for α‐SMA expression using a mouse monoclonal α‐SMA antibody (clone 1A4; Dako/Agilent Technologies, Stockport, Cheshire, UK) against its appropriate mouse isotype control IgG2a (Dako/Agilent Technologies). Primary ASM cells were used between passages 2 and 6.

The human virus‐transformed bronchial epithelial cell line BEAS‐2B cells were derived from normal bronchial epithelium obtained from autopsy of a non‐cancerous individual and purchased from the American Type Culture Collection (ATCC, Manassas, VA). BEAS‐2B cells were cultured on collagen‐coated surfaces and maintained in LHC‐9 medium (Thermo Fisher Scientific) and 1% antibiotic–antimycotic solution.

The human lung mucoepidermoid carcinoma epithelial cell line H292 was purchased from ATCC. H292 cells were maintained in RPMI 1640 medium (LGC Standards, Teddington, Middlesex) supplemented with 10% FBS and 1% antibiotic–antimycotic solution.

Mast cells were isolated from lung tissue obtained from patients undergoing lung resection for lung cancer. The study was approved by the Leicestershire Research Ethics Committee, and all tissue donors gave written informed consent. HLMCs were isolated from macroscopically normal lung tissue obtained at surgery for carcinoma by positive selection using immunomagnetic Dynabeads coated with mouse monoclonal CD117 antibody (clone YB5.B8; BD Bioscience, Berkshire) as described previously.^29^ Following HLMC purification, cells were cultured in DMEM/ GlutaMAX/HEPES (Thermo Fisher Scientific) supplemented with 10% FBS, 1% antibiotic–antimycotic solution, 1% nonessential amino acids, stem cell factor (100 ng mL^−1^ R&D Systems, Abingdon), IL‐6 (50 ng mL^−1^ R&D Systems) and IL‐10 (10 ng mL^−1^ R&D Systems). HLMC purity was > 99%, and cell viability was > 98%.

The human mast cell line HMC‐1 cells were a generous gift from Dr. J. Butterfield (Mayo Clinic, Rochester, MN). Cells were maintained in Iscove’s medium (Thermo Fisher Scientific) containing 10% iron‐supplemented foetal calf serum (Sigma‐Aldrich) and 1% antibiotic–antimycotic solution.

### Immunohistochemistry

Sections from glycomethacrylate‐embedded (2 µm) bronchial biopsies were stained using mouse monoclonal IL‐18 antibody (clone 25‐2G; MBL International, Woburn, MA), IL‐18BPa antibody (clone 136033; R&D Systems) and rabbit polyclonal IL‐18Rα antibody (Sigma‐Aldrich). Appropriate mouse isotype control IgG1 (Dako/Agilent Technologies) and rabbit isotype control (Immunostep, Salamanca) were applied respectively. The EnVision FLEX Kit (Dako/Agilent Technologies) was used for the staining, and all slides were stained on the PTLink/Autostainer Link 48 (Dako/Agilent Technologies) to assure consistency of staining. Nuclei were identified with Mayer's haematoxylin (blue staining, Sigma‐Aldrich). The quantitative assessment of IL‐18 staining in the epithelium was measured by reciprocal intensity. IL‐18BP‐ and the IL‐18Rα‐positive stainings were quantified per mm^2^ epithelial, lamina propria or smooth muscle area and evaluated using the ZEN2 software (Zeiss 2012; Cambourne, Cambridge).

### Flow cytometry

Cells were counted by trypan blue exclusion (Sigma‐Aldrich), and 95–100% viable 1 × 10^6^ cells mL^–1^ were resuspended into phosphate‐buffered saline (PBS; Sigma‐Aldrich) containing 0.5% bovine serum albumin (BSA; Sigma‐Aldrich). Cells were stained with primary antibodies against the protein of interest (mouse monoclonal IL‐18, clone 125‐2H, MBL International; IL‐18BPa, clone 136033; IL‐18Rα RPE‐conjugated, clone 70625, R&D Systems; and IL‐18Rβ RPE‐conjugated, clone 132029) versus appropriate isotype controls (mouse isotype control IgG1; mouse isotype RPE‐conjugated IgG1 isotype control (BD Bioscience); and mouse isotype RPE‐conjugated IgG2b isotype control, R&D Systems). To investigate cell differentiation/EMT changes in primary bronchial epithelial and BEAS‐2B cells, cells were activated for 24 h ± with IL‐18 (50 ng mL^−1^ MBL International) harvested and stained with primary antibodies against the protein of interest (mouse monoclonal E‐cadherin, clone 180215, R&D Systems; α‐SMA, clone 1A4; fibronectin RPE‐conjugated, clone P1H11, R&D Systems; and rabbit polyclonal collagen I, Sigma‐Aldrich) versus appropriate isotype controls (mouse isotype control IgG1; RPE‐conjugated IgG1 isotype control, R&D Systems; and rabbit isotype control). For total protein expression (cell surface and intracellular expression combined), cells were fixed and permeabilised in 4% paraformaldehyde (Thermo Fisher Scientific) and 0.1% saponin (Sigma‐Aldrich) for 15 min on ice, then labelled with appropriate cell markers. Acquisition of all cells was performed by one‐colour flow cytometry on the BD FACSCanto flow cytometer (BD Bioscience) collecting 10,000 events. The settings for the forward scatter (FSC) and side scatter (SSC) signals were adjusted so that cells of interest were clearly displayed on a dot plot using logarithmic scales of FSC versus SSC. A gate was set around the cells, as determined from the light scatter properties. Voltages were set so that over 95‐100% of unlabelled cells appeared within the first decade on histograms of fluorescence of FITC‐labelled (green) or RPE‐labelled (red) cells.

### Immunofluorescence

For immunofluorescence, epithelial cells were cultured onto collagen‐coated 25‐mm glass coverslips in 6‐well plates to subconfluence. ASM cells were cultured directly onto 8‐well chamber slides, and mast cells were cultured onto fibronectin (Sigma‐Aldrich)‐coated 8‐well slides. Cells were activated for 24 h ± IL‐18 (50 ng mL^−1^) and fixed with either methanol (Sigma‐Aldrich) or 4% formaldehyde (Sigma‐Aldrich), permeabilised with Triton X 0.15% (Sigma‐Aldrich) and blocked with 3% BSA in PBS. Slides were stained for IL‐18BPa (clone 136033), IL‐18Rα, E‐cadherin, α‐SMA, collagen I and fibronectin RPE‐conjugated antibodies with appropriate isotype control (overnight), indirectly labelled with secondary RPE or FITC (90 min, Dako/Agilent Technologies) and 4′,6‐diamidino‐2‐phenylindole (Sigma‐Aldrich) staining of cell nuclei. Cells were studied by a standard fluorescent microscope. For the quantification of immunofluorescence images, the same light intensity and gain voltage exposure time were set for the isotype control and test sample (untreated or treated). Four randomly selected areas were photographed at x40, and images were saved and measured for percentage area using ImageJ software and averaged.

### Western Blotting

Adherent cells were seeded at 1.5 x 10^5^ cells in two 60‐mm Petri dishes and grown to subconfluence, and total 3 x 10^5^ cells of suspension cell pellet were collected. Whole‐cell protein extracts were prepared by lysing the cells in Laemmli buffer, followed by sonication and boiling for 3 min. Cells were normalised for cell number. After denaturation, proteins were separated by SDS‐PAGE before wet transfer onto PVDF membrane, which were blocked with 5% milk in Tris‐buffered saline (Bio‐Rad Laboratories, Watford, Hertfordshire), containing 0.1% Tween‐20 (TBS‐T, Sigma‐Aldrich). Blocked membranes were probed with IL‐18 antibody (clone 25‐2G, 1 µg mL^−1^) or E‐cadherin (clone 180224, 2 µg mL^−1^) in TBS‐T plus 5% milk overnight at 4°C, washed several times with TBS‐T and incubated for 1 h with anti‐rabbit or anti‐mouse peroxidase‐conjugated antibody (1:1000; Dako/Agilent Technologies). Western blots were visualised using an enhanced chemiluminescence kit according to the manufacturer's instructions (GE Healthcare, Buckinghamshire). Signals were then visualised using ImageQuant LAS 4000 (GE Healthcare). The band intensity was quantified by densitometric analyses using the ImageJ software.

### Quantitative PCR

Quantitative PCR (qPCR) of IL‐18, IL‐18BP, IL‐18Rα, IL‐18β, E‐cadherin, α‐SMA, collagen I and fibronectin messenger RNAs was performed in cells. Total RNA was purified using PeqGold Total RNA Kit (VWR International, Lutterworth, Leicestershire) followed by DNaseI (VWR International) treatment. RNA quality and quantity were assessed using a Tecan infinite NANO‐QUANT plate reader (Tecan Ltd, Reading), and 2 µg of total RNA from each cell culture was reverse‐transcribed using SuperScript Vilo cDNA Synthesis Kit (Thermo Fisher Scientific). Amplification of 10 or 200 ng of cDNA per reaction in a final volume of 20 µL was performed using the Express SYBR GreenER qPCR SuperMix Universal (Thermo Fisher Scientific) in a 7900HT Fast Real‐Time PCR System (Thermo Fisher Scientific). After an initial incubation for 2 min at 50°C followed by 5 min at 95°C, the conditions of amplification were as follows: denaturation at 95°C, annealing at 60°C and extension at 72°C for 40 cycles. 18S or GAPDH rRNA was used as reference genes. The expression data for each transcript were presented relative to the reference gene and determined using the equation 2^−^dCq, where dCq = (Cq_target gene_ – Cq_18S RNA_), and this expression was arbitrarily multiplied by the factor 10^6^ for clearer presentation. Relative expression of the mRNA encoding E‐cadherin, α‐SMA, collagen I and fibronectin was normalised to GAPDH mRNA expression. Details of the qPCR primers used in this study are listed in Supplementary table 1.

### Calcium flux

Cells were labelled in parallel with 10 µg mL^−1^ fluo‐3 and 4 µg mL^−1^ Fura Red acetoxymethyl esters (Thermo Fisher Scientific) for 45 min at 37°C in phosphate saline solution + 2 mm Ca^2+^. For determination of cellular response, baseline unstimulated measurements (1 min) were followed by addition of IL‐18 (50–200 ng mL^−1^) or calcium ionophore (1.5 µg mL^−1^; Sigma‐Aldrich) as a positive control. The cell flow was halted during this addition, appended and then continued acquiring for a further minute. Data were collated in a histogram displaying the ratio of Fluo‐3/Fura Red versus time. The geometric mean fluorescence intensity (GMFI) of the unstimulated cell population was compared with the stimulated population.^30^, ^31^

### Wound repair

Primary bronchial epithelial cells were seeded onto 6‐well plates coated with collagen until 80–90% confluence. Cells were wounded using a sterile 200‐µL pipette tip in a predetermined grid pattern.^30^ Cells were then washed prior to addition of fresh media ± IL‐18 (50 ng mL^−1^), IL‐18BP (150 ng mL^−1^, R&D Systems) and TGF‐β1 (10 ng mL^−1^, R&D Systems) as a positive control. Wounds were photographed at baseline and after 24 h. The percentage of cells that had moved into the wound was analysed using photoshop CC software.

### Semiquantitative assessment of cell morphology and cell counts

Human primary bronchial epithelial cells and BEAS‐2B cells were seeded in 60‐mm Petri dishes at a density of 1.5 × 10^5^ cells per dish and left to reach 80% confluence. The cells were then treated/untreated with 50 ng mL^−1^ of IL‐18 for 24 h respectively. Four random fields for each Petri dish were then photographed after 24 h. Total cell counts were conducted in each of the four random fields, and the number of elongated/spindle‐like shaped cells was counted. The cells were then harvested with trypsin (Sigma‐Aldrich) and counted using trypan blue solution used as a cell viability dye.

### Microarray platform

Total RNA purified from primary bronchial epithelial cells and ASM treated and untreated with IL‐18 (50 ng mL^−1^) for 6 h followed by DNaseI treatment and RNA integrity was checked on a Bioanalyzer 2100 (Dako/Agilent Technologies). RNA was converted to cDNA and then to cRNA using Illumina (Illumina, San Diego, CA) TotalPrep™ RNA Amplification Kit according to the manufacturer’s instructions (Thermo Fisher Scientific). Whole‐transcriptome expression profiling was performed on each sample using Illumina HumanHT‐12 v4 Expression BeadChips, according to the manufacturer’s protocol. The GenomeStudio analytical software was used to assess the raw data quality, perform quantile normalisation and determine the average background intensity. Probes with signal intensity below the background were excluded from subsequent analyses.^30^

### Mesoscale analysis of supernatants from ASM and HMC‐1 cells

The concentration of a panel of cytokines and chemokines was measured in ASM, HMC‐1 cells and HLMC ± IL‐18 (10 ng mL^−1^ or 100 ng mL^−1^) for 24 h by electrochemiluminescence detection and pattern arrays (Meso Scale Diagnostics LLC, Rockville, MD). The panel included the cytokines (ChTr, EGF, huYKL‐40, IL‐1α, IL‐1β, IL‐2, IL‐6, IL‐6R, IL‐8, IL‐9, IL‐12 p70, IL‐13, IL‐15, IL‐23, IL‐25, IL‐33, C‐X‐C motif chemokine (CXCL)10, matrix metalloproteinase‐9, nerve growth factor, stem cell factor, interleukin‐1 receptor, tumor necrosis factor α, TNF receptor 1, TNF receptor 11, thymic stromal lymphopoietin, vascular endothelial growth factor (VEGF) and chemokines C‐C motif chemokine (CCL)2, CCL4, CCL5, CCL11, CCL17, CCL26 and CXCL9, CXCL11 and CXCL17). The limit of detection of the mesoscale system was 2.4 pg mL^−1^ for all cytokines and chemokines.

### ELISA

The concentrations of IL‐18 (Thermo Fisher Scientific) and IL‐18BPa (R&D Systems) were quantified in patient‐induced sputum samples and cell supernatants by ELISA according to the manufacturer’s instructions. The sensitivity limit of the ELISAs was as follows: IL‐18, 78 pg mL^−1^, and IL‐18BPa, 93.8 pg mL^−1^.

### Apoptosis

The percentage of apoptotic BEAS‐2B cells ± IL‐18 (100 ng mL^−1^) or IL‐18BP (150 ng mL^−1^ after treated for 24 h) was identified by staining with FITC‐conjugated Annexin V (1 µL/200 µL binding buffer, BD Bioscience) ± propidium iodide (PI, 0.5 μg mL^−1^, BD Bioscience). Acquisition of all cells was performed by two‐colour flow cytometry on the BD FACSCanto flow cytometer (BD Bioscience) collecting 10 000 events.

### Cell metabolic activity assay

The CellTiter 96^®^ AQueous One solution Assay Kit with the tetrazolium compound (3‐(4,5‐dimethylthiazol‐2‐yl)‐5‐(3‐carboxymethoxyphenyl)‐2‐(4‐sulfophenyl)‐2H) (MTS; Promega, Chilworth, Southampton) was used to measure the metabolic activity of cells seeded at 3 × 10^3^ per well into a 96‐well plate ± IL‐18 (12.5–100 ng mL^−1^) or IL‐18BP (150 ng mL^−1^). The cells were treated for 24 h; thereafter, a CellTiter 96^®^ AQueous assay was conducted following the manufacturer’s instructions.

### CyQUANT^®^ cell proliferation assay

Proliferation of BEAS‐2B cells was assayed using the CyQUANT® kit (Thermo Fisher Scientific). Cells were seeded at 3 x 10^3^ per well into a 96‐well plate ± IL‐18 (50 ng mL^−1^) and IL‐18BP (150 ng mL^−1^). The cells were stimulated for 24 h, washed and frozen at –20°C. Total DNA was then quantified following the manufacturer’s instructions. The fluorescence of samples was measured with excitation at 485 nm and emission detection at 535 nm using the EnVision plate reader (PerkinElmer Ltd, Ynysmaerdy, Pontyclun).

### Assessment of cell contraction by collagen gel analysis

A total of 2.5 × 10^5^ ASM cells were resuspended in 144 μL DMEM with GlutaMAX‐1 serum‐free media, with 299 μL collagen, 37 μL 10X DMEM (Thermo Fisher Scientific) and 20 μL sodium bicarbonate (Thermo Fisher Scientific) and added to 24‐well plates (precoated with BSA 2% overnight in a CO^2^ incubator set at 37°C). The mixture was left to polymerise into gels at 37°C for 90 min prior to detachment from the well, and 500 μL of media ± stimulus was added and incubated over 3 days. Gel surface area was measured using the ImageJ software.

### Histamine assay

HLMCs were activated using the high‐affinity IgE receptor (FcεR1) antibody (1:1000; Millipore, Ltd, Watford), IL‐18 or IL‐18BP at 10–100 ng mL^−1^ for 24 h and supernatants collected for histamine release. Histamine was measured by sensitive radioenzymatic assay based on the conversion of histamine to methylhistamine in the presence of the enzyme histamine‐N‐methyltransferase as previously described.^32^

### Statistical analysis

Statistical analysis was performed using PRISM version 7 (La Jolla, California). Data are presented as mean or median (± SEM). Nonparametric data were analysed using the Mann–Whitney *U‐*test and the parametric data by using *t*‐tests and analysis of variances. Differences were considered significant when *P*‐values were  < 0.05. For microarray data, raw fluorescence signal intensities of each Illumina probe were averaged across all samples. Probes with an average raw signal intensity across the samples below the confidence detection level determined by the GenomeStudio control panel were considered absent. Those considered present raw signal intensities were log_2_‐transformed, and transcripts significantly differentially expressed between untreated and IL‐18‐treated cells were determined using a two‐tailed unpaired Student’s *t*‐test in Microsoft Excel. Transcripts were then ranked according to the *P*‐value and fold changes. Transcripts with a *P*‐value < 0.1 and a fold difference of 2 between groups were selected and taken forward.

## Conflict of interest

CEB received grants from Airway Disease Predicting Outcomes through Patient Specific Computational Modelling (AirPROM) project (funded through FP7 EU grant), NC3R and the European Regional Development Fund (ERDF 05567) part‐funded the research laboratories. This paper presents independent research funded by the National Institute for Health Research (NIHR). The views expressed are those of the authors and not necessarily those of the NHS, the NIHR or the Department of Health. DK, LC, EG and NS have no conflicts of interest in relation to this work.

## Author contribution

**Davinder Kaur:** Conceptualization; Data curation; Formal analysis; Investigation; Methodology; Project administration; Resources; Software; Validation; Visualization; Writing‐original draft; Writing‐review & editing. **Latifa Chachi:** Formal analysis; Methodology; Visualization; Writing‐review & editing. **Edith Gomez:** Formal analysis; Methodology; Writing‐review & editing. **Nicolas Sylvius:** Formal analysis; Methodology; Writing‐review & editing. **Christopher E Brightling:** Conceptualization; Data curation; Formal analysis; Funding acquisition; Investigation; Project administration; Supervision; Validation; Writing‐original draft; Writing‐review & editing.
