# Supplementary material for: Interleukin‐18, IL‐18 binding protein and IL‐18 receptor expression in asthma: a hypothesis showing IL‐18 promotes epithelial cell differentiation
Source: Clin Transl Immunology. 2021 Jun 26;10(6):e1301. doi: 10.1002/cti2.1301 (PMC8234286; doi:10.1002/cti2.1301)
Supplement: Supplementary file 1 [file CTI2-10-e1301-s001.pdf]

Supporting information

Interleukin-18, IL-18 binding protein and IL-18 receptor expression in asthma: with IL-18 promoting epithelial cell differentiation.

Davinder Kaur[1], Latifa Chachi[1], Edith Gomez[1], Nicolas Sylvius[2], Christopher E Brightling[1]

Affiliations:

[1]Institute for Lung Health, NIHR Biomedical Research Centre, Department of Respiratory Sciences, University of Leicester, Leicester, UK.

[2]Genomic Core Facility, Department of Genetics, University of Leicester, Adrian Building, University Road, G23, Leicester LE1 7RH, UK.

Author for Correspondence:

Professor CE Brightling, University of Leicester, Glenfield General Hospital, Leicester, LE3 9QP, U.K. Tel: 0044 116 258 3998, Fax: 0044 1162502787, E-mail: ceb17@le.ac.uk

Running head: IL-18, IL-18BP and IL-18R in human epithelium.

SUPPLEMENTARY TABLE LEGENDS

Supplementary table 1: Sequences of specific primers used in this study.

SUPPLEMENTARY FIGURE LEGENDS

Supplementary figure 1. Bronchial biopsies IL-18BP expression in ASM cells.

(a) Representative photomicrograph of normal biopsy specimen illustrating isotype control (magnification x400), IL-18BP staining within the ASM cells (original magnification x400 and x600). **(b)** Cell counts/mm^2 for IL-18BP positive cells within the ASM. Symbol key: ● = healthy control (*n* = 5); □ = mild/moderate asthma (*n* = 6); ■ = severe asthma (*n* = 8). Horizontal bars represent median, unpaired two tailed non-parametric *t*-test.

Supplementary figure 2. Expression of IL-18 and IL-18BP in human cells.

(a) Example fluorescent histograms of IL-18 and **(b)** quantification of total IL-18 in ASM (*n* = 11), H292 cells (*n* = 5), HLMC (n=3) and HMC-1 cells (*n* = 6). **(c)** Western blot analysis of IL-18 in ASM cells (*n* = 3), H292 cells (*n* = 3), HLMC (*n* = 3) and HMC-1 cells (*n* = 3, upper gel) and β-actin was used as a loading control (lower gel). **(d)** IL-18BP expression in ASM, HLMC and HMC-1 cells confirmed by immunofluorescence (nuclei stained blue), IL-18BP (stained green), isotype control shown as insert, (magnification x400 *n* = 3). **(e)** Example fluorescent histograms of IL-18BP and **(f)** quantification of total IL-18BP in ASM (*n* = 12), H292 cells (*n* = 5), HLMC (*n* = 7) and HMC-1 cells (*n* = 3). **(g)** IL-18BP spontaneous release after 24 hours in ASM (*n* = 13) and H292 cells (*n* = 3). **(h)** IL-18 and **(i)** IL-18BP mRNA expression analyzed by qPCR in ASM, HLMC and HMC-1 cells. Data are presented

as mean ± SEM. Statistical differences were assessed using the paired or unpaired *t*-test $^{**}P$ value <0.01, $^{***}P$ < 0.001 (verses isotype control).

Supplementary figure 3. Original western blot for epithelial cell IL-18 expression.

Supplementary figure 4. IL-18 receptor expression by human cells.

(a) IL-18Rα expression in ASM, HLMC and HMC-1 cells was confirmed by immunofluorescence isotype control, (nuclei stained blue), IL-18Rα (stained red) (magnification x200 n = 3). **(b)** Example fluorescent histograms of IL-18Rα and **(c)** quantification of surface IL-18Rα in ASM cells (n = 11), H292 cells (n = 3), HLMC (n = 7) and HMC-1 cells (n = 4). **(d)** Example fluorescent histograms of IL-18Rβ and **(e)** quantification of surface IL-18Rβ ASM cells (n = 3), H292 cells (n = 5), HLMC (n = 6) and HMC-1 cells (n = 7). IL-18Rα **(f)** and IL-18Rβ **(g)** mRNA expression analyzed by qPCR in ASM, HLMC and HMC-1 cells. Data are presented as mean ± SEM. Statistical differences were assessed using the paired or unpaired *t*-test $^{*}P$ value < 0.05, $^{**}P$ value < 0.01, $^{***}P$ <0.001 (verses isotype control).

Supplementary figure 5. Wound healing response IL-18 in H292 cells.

(a) Representative wound repair pictures of H292 cells after 24 hours ± IL-18 (x100 magnification). **(b)** H292 cells ± IL-18 (50 ng mL^{-1}) or ± IL-18BP (150 ng mL^{-1}) for 24 hours (n = 3). Data are presented as mean ± SEM. Statistical differences were assessed using the paired *t*-test.

Supplementary figure 6. Functional responses of IL-18 by human cells.

Metabolic activity following 24 hour exposure ± IL-18 in ASM cells **(a)** FBS and **(b)** insulin/transferrin-sodium selenite (ITS) media using the MTS assay (*n* = 5). **(c)** Concentration-dependent chemotaxis in ASM towards IL-18 (*n* = 9) following 24 hour exposure. **(d)** Collagen gel contraction in ASM cells primed with IL-18 (10 ng mL^{-1}) or bradykinin (1nM) impregnated in collagen gels for 3 days (n=3-6). (**e**) Histamine release in HLMC stimulated with anti-FcεR1 (1:1000), IL-18 (10-100 ng mL^{-1}) or IL-18BP (10 - 100 ng mL^{-1}) for 24 hours (*n* = 6-12). All data are presented as mean ± SEM. Statistical differences were assessed using the paired or unpaired *t*-test.

Supplementary figure 7. Original western blot for epithelial cell E-cadherin expression.

Supplementary table 1: Sequences of specific primers used in this study.

Gene	Forward sequence (5’ to 3’)	Reverse sequence (5’ to 3’)	Length (bp)
IL-18	TTGTCTCCCAGTGCATTTTG	CAGCAGCCATCTTTATTCCTG	166
IL-18BP	CCATTCTCTCTCCACCTATCCA	GCAAGGCTAAGGCATCAACA	100
IL-18Rα	TTGGAGAAACATTTTGGGTATAAGT	ACAATGATTAGTCTTCGGCTTTTC	116
IL-18Rβ	AAACACTCTACTCTGGCAAAGG	CAAAGAAATATCCAGCCCAAACA	91
E-cadherin Primer Design	AGACAGTGATTGAATACAAAACCA	GGAGTTTACAGGAAGCAGACA	130
α-SMA	TTCAATGTCCCAGCCATGTA	GAAGGAATAGCCACGCTCAG	222
Collagen I Primer Design	AGACAGTGATTGAATACAAAACCA	GGAGTTTACAGGAAGCAGACA	130
Fibronectin Primer Design	AGTGCTTCATGCCTTTAGATGTAC	CATGCTTGTTCCTCTGGATTGG	84
18S	GTTGGTTTTCGGAACTGAGG	GCATCGTTTATGGTCGGAAC	200
GAPDH	TGCACCACCAACTGCTTAGC	GGCATGGACTGTGGTCATGA	87

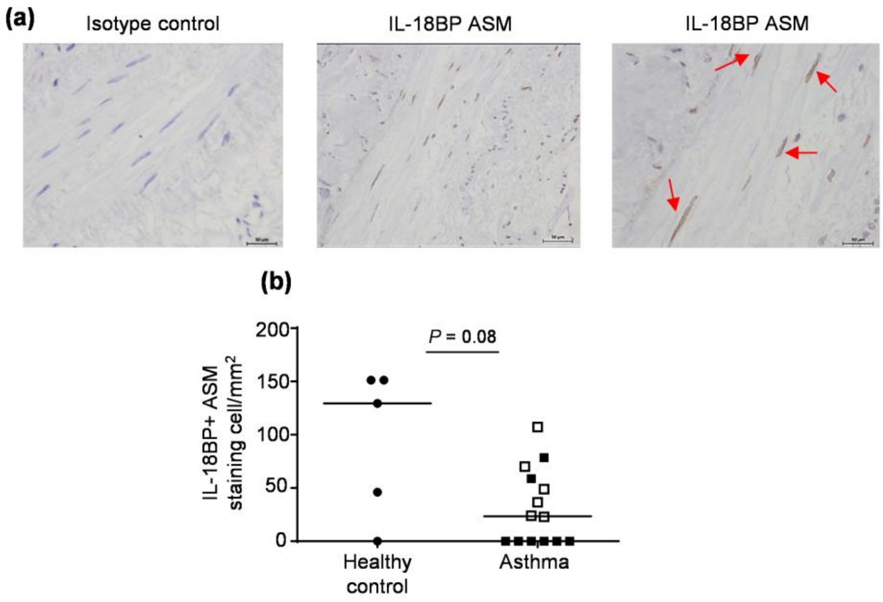

Supplementary figure 1. Bronchial biopsies IL-18BP expression in ASM cells.
(a) Representative photomicrograph of normal biopsy specimen illustrating isotype control (magnification x400), IL-18BP staining within the ASM cells (original magnification x400 and x600). **(b)** Cell counts/mm^2 for IL-18BP positive cells within the ASM. Symbol key: ● = healthy control (n = 5); □ = mild/moderate asthma (n = 6); ■ = severe asthma (n = 8). Horizontal bars represent median, unpaired two tailed non-parametric t-test.

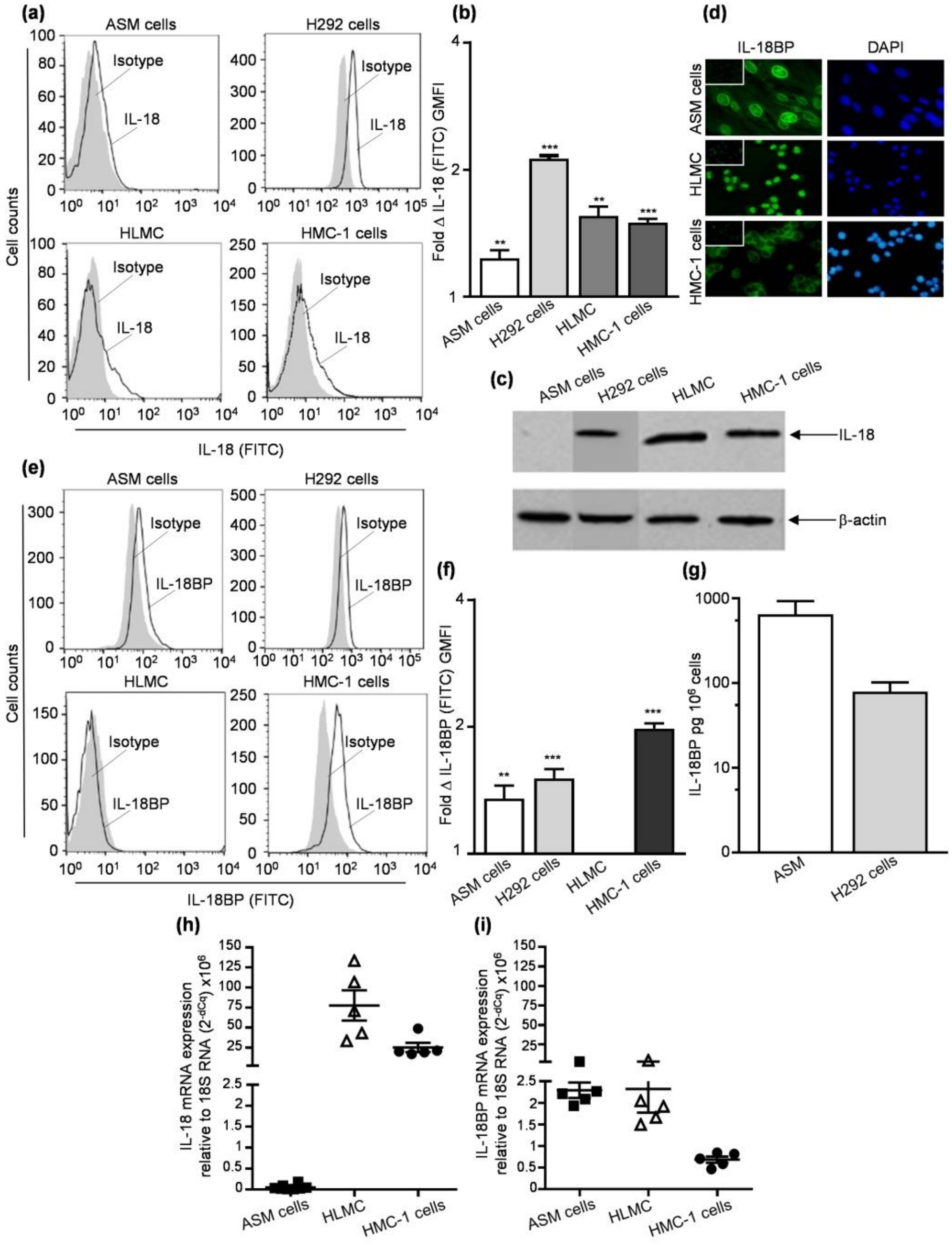

Supplementary figure 2. Expression of IL-18 and IL-18BP in human cells.
(a) Example fluorescent histograms of IL-18 and **(b)** quantification of total IL-18 in ASM (*n* = 11), H292 cells (*n* = 5), HLMC (*n* = 3) and HMC-1 cells (*n* = 6). **(c)** Western blot analysis of IL-18 in ASM cells (*n* = 3), H292 cells (*n* = 3), HLMC (*n* = 3) and HMC-1 cells (*n* = 3, upper gel) and β-actin was used as a loading control (lower gel). **(d)** IL-18BP expression in ASM, HLMC and HMC-1 cells confirmed by immunofluorescence (nuclei stained blue), IL-18BP (stained green), isotype control shown as insert, (magnification x400 *n* = 3). **(e)** Example fluorescent histograms of IL-18BP and **(f)** quantification of total IL-18BP in ASM (*n* = 12), H292 cells (*n* = 5), HLMC (*n* = 7) and HMC-1 cells (*n* = 3). **(g)** IL-18BP spontaneous release after 24 hours in ASM (*n* = 13) and H292 cells (*n* = 3). **(h)** IL-18 and **(i)** IL-18BP mRNA expression analyzed by qPCR in ASM, HLMC and HMC-1 cells. Data are presented as mean ± SEM. Statistical differences were assessed using the paired or unpaired *t*-test $^{**}P$ value <0.01, $^{***}P < 0.001$ (verses isotype control).

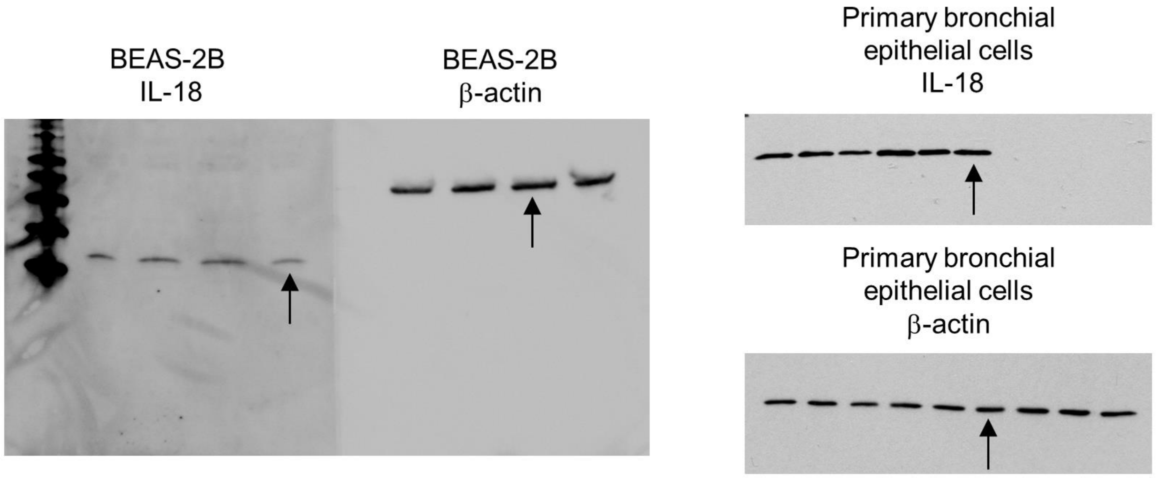

Supplementary figure 3. Original western blot for epithelial cell IL-18 expression.

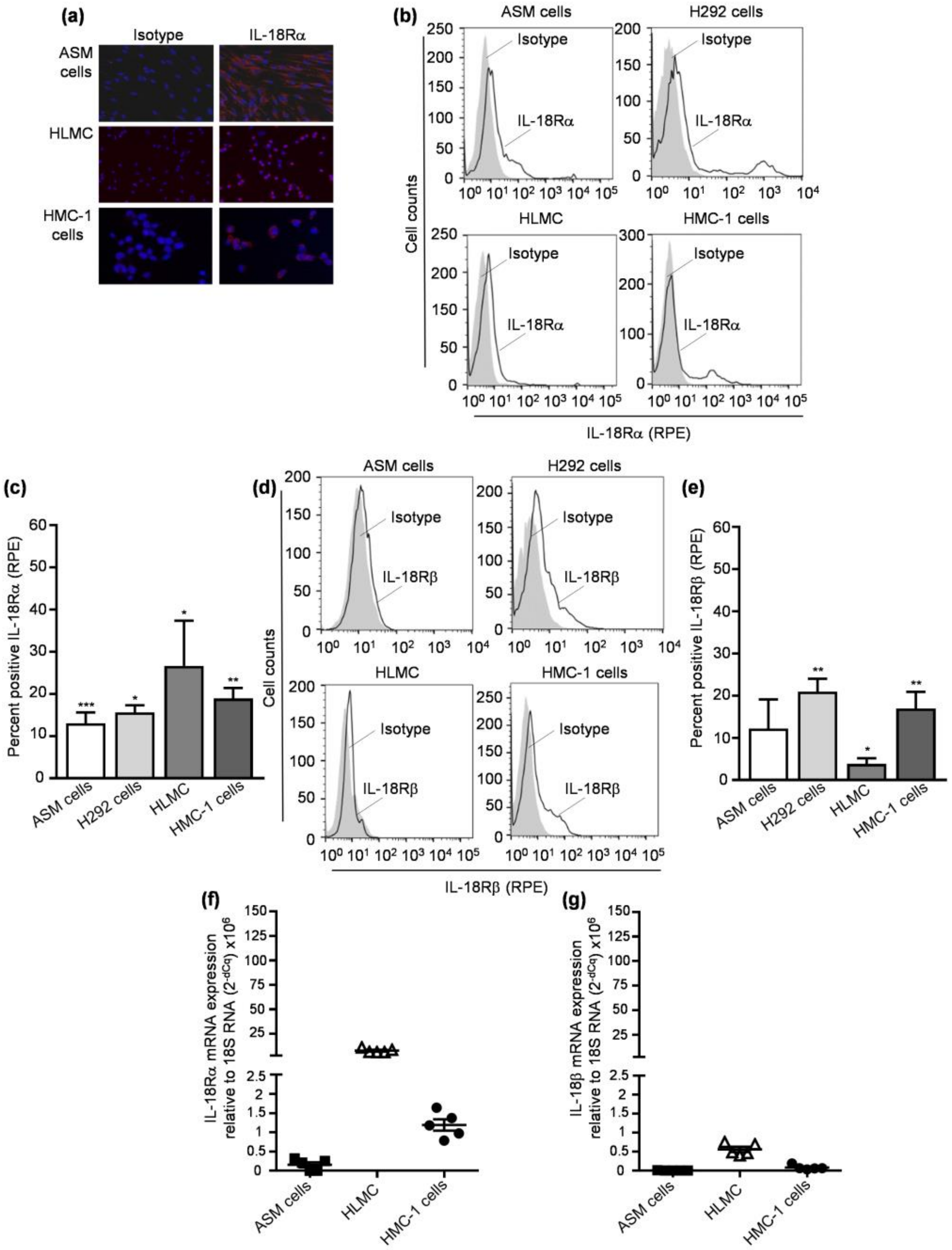

Supplementary figure 4. IL-18 receptor expression by human cells.
(a) IL-18Rα expression in ASM, HLMC and HMC-1 cells was confirmed by immunofluorescence isotype control, (nuclei stained blue), IL-18Rα (stained red) (magnification x200 n = 3). **(b)** Example fluorescent histograms of IL-18Rα and **(c)** quantification of surface IL-18Rα in ASM cells (n = 11), H292 cells (n = 3), HLMC (n = 7) and HMC-1 cells (n = 4). **(d)** Example fluorescent histograms of IL-18Rβ and **(e)** quantification of surface IL-18Rβ ASM cells (n = 3), H292 cells (n = 5), HLMC (n = 6) and HMC-1 cells (n = 7). IL-18Rα **(f)** and IL-18Rβ **(g)** mRNA expression analyzed by qPCR in ASM, HLMC and HMC-1 cells. Data are presented as mean ± SEM. Statistical differences were assessed using the paired or unpaired t-test *P value < 0.05, **P value < 0.01, ***P < 0.001 (verses isotype control).

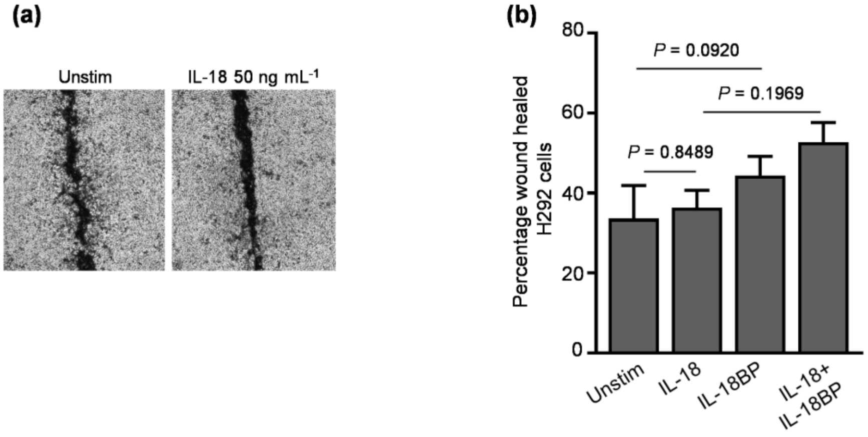

Supplementary figure 5. Wound healing response IL-18 in H292 cells.
(a) Representative wound repair pictures of H292 cells after 24 hours ± IL-18 (x100 magnification). **(b)** H292 cells ± IL-18 (50 ng mL^{-1}) or ± IL-18BP (150 ng mL^{-1}) for 24 hours (*n* = 3). Data are presented as mean ± SEM. Statistical differences were assessed using the paired *t*-test.

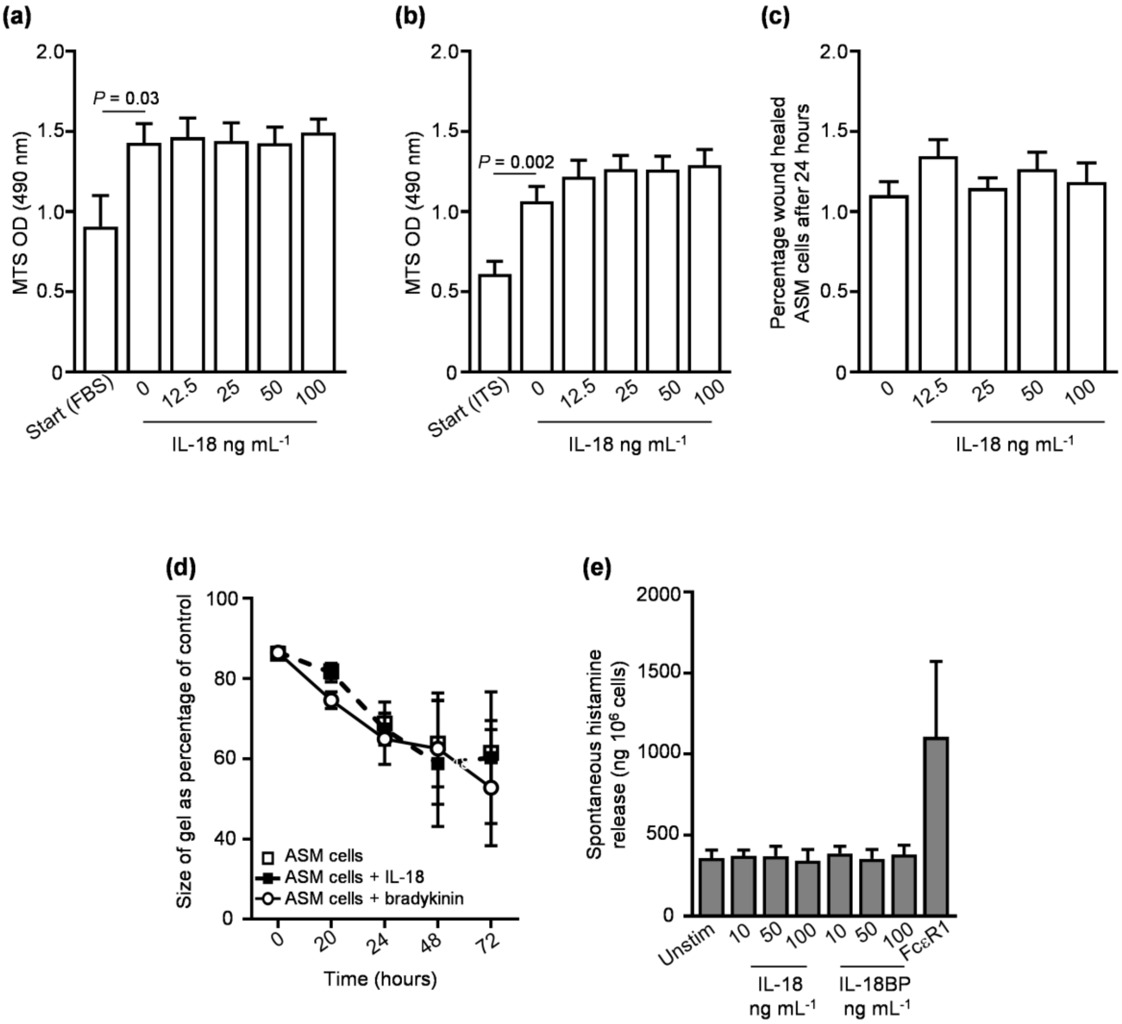

Supplementary figure 6. Functional responses of IL-18 by human cells.
Metabolic activity following 24 hour exposure ± IL-18 in ASM cells **(a)** FBS and **(b)** insulin/transferrin-sodium selenite (ITS) media using the MTS assay (*n* = 5). **(c)** Concentration-dependent chemotaxis in ASM towards IL-18 (*n* = 9) following 24 hour exposure. **(d)** Collagen gel contraction in ASM cells primed with IL-18 (10 ng mL^{-1}) or bradykinin (1nM) impregnated in collagen gels for 3 days (*n* =3-6). (**e**) Histamine release in HLMC stimulated with anti-FcεR1 (1:1000), IL-18 (10-100 ng mL^{-1}) or IL-18BP (10 - 100 ng mL^{-1}) for 24 hours (*n* = 6-12). All data are presented as mean ± SEM. Statistical differences were assessed using the paired or unpaired *t*-test.

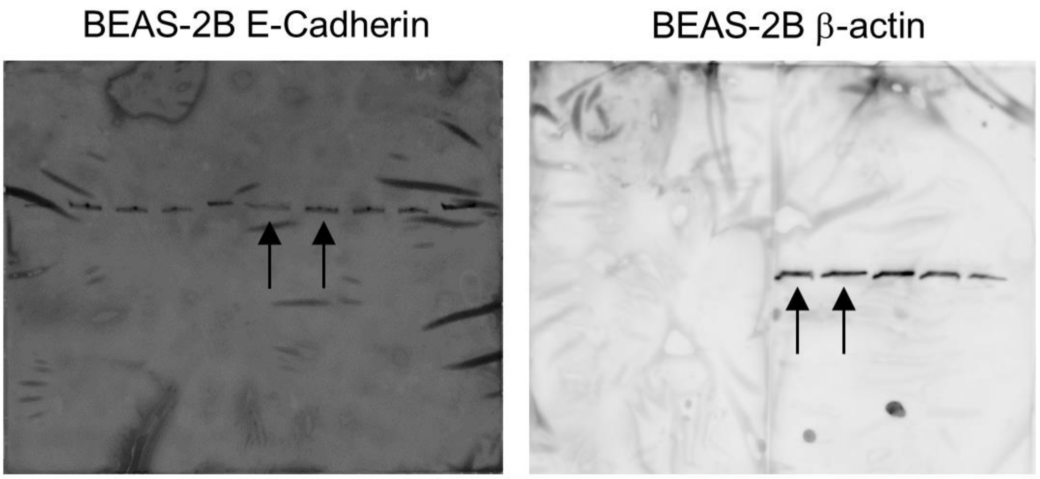

Supplementary figure 7. Original western blot for epithelial cell E-cadherin expression.
